# Bacterial and fungal communities in indoor aerosols from two Kuwaiti hospitals

**DOI:** 10.3389/fmicb.2022.955913

**Published:** 2022-07-28

**Authors:** Nazima Habibi, Saif Uddin, Montaha Behbehani, Fadila Al Salameen, Nasreem Abdul Razzack, Farhana Zakir, Anisha Shajan, Faiz Alam

**Affiliations:** Environment and Life Science Research Centre, Kuwait Institute for Scientific Research, Kuwait City, Kuwait

**Keywords:** bioaerosol, bacteria, fungi, virus, exhaled air

## Abstract

The airborne transmission of COVID-19 has drawn immense attention to bioaerosols. The topic is highly relevant in the indoor hospital environment where vulnerable patients are treated and healthcare workers are exposed to various pathogenic and non-pathogenic microbes. Knowledge of the microbial communities in such settings will enable precautionary measures to prevent any hospital-mediated outbreak and better assess occupational exposure of the healthcare workers. This study presents a baseline of the bacterial and fungal population of two major hospitals in Kuwait dealing with COVID patients, and in a non-hospital setting through targeted amplicon sequencing. The predominant bacteria of bioaerosols were *Variovorax* (9.44%), *Parvibaculum* (8.27%), *Pseudonocardia* (8.04%), *Taonella* (5.74%), *Arthrospira* (4.58%), *Comamonas* (3.84%), *Methylibium* (3.13%), *Sphingobium* (4.46%), *Zoogloea* (2.20%), and *Sphingopyxis* (2.56%). ESKAPEE pathogens, such as *Pseudomonas*, *Acinetobacter*, *Staphylococcus*, *Enterococcus*, and *Escherichia*, were also found in lower abundances. The fungi were represented by *Wilcoxinia rehmii* (64.38%), *Aspergillus ruber* (9.11%), *Penicillium desertorum* (3.89%), *Leptobacillium leptobactrum* (3.20%), *Humicola grisea* (2.99%), *Ganoderma sichuanense* (1.42%), *Malassezia restricta* (0.74%), *Heterophoma sylvatica* (0.49%), *Fusarium proliferatum* (0.46%), and *Saccharomyces cerevisiae* (0.23%). Some common and unique operational taxonomic units (OTUs) of bacteria and fungi were also recorded at each site; this inter-site variability shows that exhaled air can be a source of this variation. The alpha-diversity indices suggested variance in species richness and abundance in hospitals than in non-hospital sites. The community structure of bacteria varied spatially (ANOSIM *r*^2^ = 0.181–0.243; *p* < 0.05) between the hospital and non-hospital sites, whereas fungi were more or less homogenous. Key taxa specific to the hospitals were Defluvicoccales, fungi, Ganodermataceae, *Heterophoma*, and *H. sylvatica* compared to Actinobacteria, *Leptobacillium*, *L. leptobacillium*, and Cordycipitaceae at the non-hospital site (LefSe, FDR q ≤ 0.05). The hospital/non-hospital MD index > 1 indicated shifts in the microbial communities of indoor air in hospitals. These findings highlight the need for regular surveillance of indoor hospital environments to prevent future outbreaks.

## Introduction

With the outbreak of COVID-19 and evidence of pandemic spread ***via*** air droplets, bioaerosol research has picked up momentum (**Babin, 2020**; **Faridi et al., 2020**; **Lednicky et al., 2020**; **Li et al., 2020**; **Liu et al., 2020**; **Santarpia et al., 2020**; **Tan et al., 2020**; Van Doremalen et al., 2020; **Zhou et al., 2020**; Habibi et al., 2021a,c, d, 2022a; **Li, 2021**; **Tellier, 2022**). A diverse microbial assemblage, including bacterial, fungal, and viral communities and pollens, is an integral part of bioaerosols (**Prussin and Marr, 2015**; **Prussin et al., 2015**; Habibi et al., 2022b). Studies have shown the prevalence of SARS-CoV-2 co-infection with non-coronavirus respiratory pathogens (**Kim et al., 2020**; **Abbas et al., 2021**; **Habibi et al., 2021d**; **Karimzadeh et al., 2021**). Hospitals being critical infrastructure for patient care, house patients with the compromised immune system and higher susceptibility to infection are at higher risk (**Macintyre et al., 2014**; **Stockwell et al., 2019**). Hospital-mediated outbreaks are not new (O’connor et al., 2015; **Lax et al., 2017**; **Park et al., 2018**; **Weber et al., 2019**; **Mendes et al., 2022**). More recently, hospitals were the hot spots for the novel SARS-CoV-2 spread through airborne transmission (**Duverger et al., 2021**; **Løvestad et al., 2021**; **Paltansing et al., 2021**), and reports of individuals involved in patient care getting infected and reinfected were not uncommon. Hence, characterization of the indoor aerosols is vital, as these microbes enter the human respiratory system leading to both chronic responses due to prolonged exposure and acute responses upon inhalation in case of compromised immune systems. Microbial inhalation may cause hypersensitive responses, asthma, allergic reactions, pulmonary infections, and toxicosis (**Jiayu et al., 2019**; Gevao et al., 2013, 2022).

However, the low concentration of microbial load in aerosol makes their identification an arduous task (Gregson et al., 2021; Uddin et al., 2022) that often does not get immediate attention. The microbial identification becomes further challenging due to matrix interference, physical damage, and crumbling due to the high-pressure collection of aerosol samples (Fennelly et al., 2015; Yao et al., 2021). Next-generation sequencing (NGS) has provided an effective solution for providing information on the bacterial, fungal, and viral communities in samples with high precision and accuracy (Chakrawarti et al., 2020; Habibi et al., 2021b). Next-generation sequencing has an advantage over the conventional culture-based microbial identification method that neglects the important indoor microorganisms that develop a viable but non-cultivable state (Muthuirulan and Sharma, 2017; Tong et al., 2017; Gao et al., 2018; He et al., 2020). The 16s amplicon sequencing method has been applied for bacterial community profiling from indoor aerosols (Chen et al., 2017; Chakrawarti et al., 2020; Behbehani et al., 2021a; Lee et al., 2021), particulate matter in the air (Habibi et al., 2021e), and dust (Al Salameen et al., 2020; Li et al., 2021), among other matrixes. The targeted sequences of the internal transcribed spacer (ITS) region have also been sequenced to gain information on the fungal communities within air samples (Okten and Asan, 2012; Al Salameen et al., 2020; Behbehani et al., 2021a,b; Lee et al., 2021; Salameen et al., 2021). Additionally, the 16S rRNA gene studies have extended our understanding of the functional contribution of individual community members (Shobo et al., 2020).

Besides the fact that the dispersal of microbial communities between humans and the surrounding environment through airborne release is a key medium to intercede outbreaks, the role of bioaerosols in the spread of pathogens remains largely unexplored in the region. Predominant viral communities were identified in the Kuwait’s hospital during the COVID-19 pandemic (Habibi et al., 2021b,e,g,2022a). Bacterial and fungal cells were also present, suggesting their roles in the spread of respiratory pathogens to other locations (Habibi et al., 2021a,c). This triggered an interest to map the bacterial and fungal genera through high-throughput molecular studies. The current study was aimed at an assessment of the indoor aerosol in two major hospitals (Mubarak Al Kabeer and Sheikh Jaber) in Kuwait involved in COVID-19 patient care and a non-hospital location. The characterization of bacterial communities was done using 16s rRNA gene and fungal communities using ITS region sequencing. The baseline microbial diversity in indoor air was compared between sites. The findings will improve the understanding of the bacterial and fungal populations present within the confined spaces in hospitals and aid to formulate strategies to protect healthcare personnel and uninfected people involved in patient care.

## Materials and methods

### Sampling site and aerosol collection

The indoor aerosol samples were collected between August and October 2020 amidst the height of COVID-19 lockdown after obtaining permission from the Ministry of Health, Kuwait. The aerosol samples were collected from Mubarak Al Kabeer (MKH) and Sheikh Jaber (SJH), involved in treating COVID-19 patients in Kuwait City and in a non-hospitalized setting at the Kuwait Institute for Scientific Research (KISR). Additional details are provided in Table 1. A custom-made sampling device was used to collect ambient air samples owing to the ongoing pandemic; aerosol was drawn at 30 L min^–1^ for 2 h collecting 3.6 m^3^ of aerosol (Habibi et al., 2021a). The collected bioaerosols were concurrently lysed while sampling to avoid SARS-CoV-2 dissemination. Trizol was used as a lysis and transport reagent to the laboratories to extract nucleic acid under BSL2 cabinets.

**TABLE 1:** Description of sampling locations.

Sampling site	Group	GPS coordinates	Number of sampling points	Description of sampling points
Mubarak Al Kabeer Hospital	H1	29.3260° N, 48.0350° E	6	Samples were collected near the main entrance, Casualty reception, pediatric casualty, central laboratories, pharmacy, and COVID ward
Sheikh Jaber Hospital	H2	29.2768° N, 48.0063° E	4	Samples collected from COVID, isolation area, COVID ward, Virology laboratory, and Cytology laboratory
Kuwait Institute for Scientific Research	G2	29.3369° N, 47.9064° E	6	Samples were collected from the main reception, areas near the lift, areas near the attendance recorder, location in the laboratory
Total no of samples			16	

### RNA extraction and cDNA conversion

The standard procedure of nucleic acid isolation from Trizol™ reagent (APB, Biosciences, Rockville, MD, United States) was followed to purify total RNA (Habibi et al., 2021d). The aerosols collected in Trizol™ were left at room temperature for 10 min, and the crude RNA was separated by adding 0.2 volumes of chloroform (Sigma Aldrich, WGK, Germany). The aqueous chloroform layer was transferred to 0.5 volumes of isopropanol (Merck, Darmstadt, Germany) to precipitate the RNA and subsequently washed with 70% ethanol (Merck, Rahway, NJ, United States) twice. The procedure is described in detail by Habibi et al. (2021a). The RNase-free water (Ambion, Austin, TX, United States) was used to dissolve the air-dried pellet. The high-sensitivity Qubit, HS ssRNA kit, was used to isolate RNA. The fluorometric estimation of isolated RNA was performed on a Qubit 4 Fluorometer (Thermo Scientific). The extracted total RNA was converted to complementary deoxyribonucleic acid (cDNA) using iScript™ Reverse Transcription Supermix (Bio-Rad, Darmstadt, Germany) for bacterial and fungal sequencing. The reaction mix was assembled by adding 18 μl of master mix to 2 μl of RNA and incubated for 5 min at 20°C (priming), 20 min at 46°C (revere transcription), and 1 min at 95°C (RT inactivation) (Li et al., 2017).

### Targeted amplicon (16S rDNA/ITS) sequencing

The cDNA was used for PCR amplification by employing 16Sr RNA primers (515F-806R) targeting the V4 region of bacteria and 18S rRNA primers (528F-706R) targeting the internal transcribed spacer (ITS1 and ITS 2) region of fungi (Li et al., 2017). Phusion High-Fidelity PCR Master Mix (New England Biolabs) was used for carrying out all PCR reactions. The libraries were generated with NEBNext UltraTM DNA Library Prep Kit (New England BioLabs, France). Fluorometry (Qubit, Invitrogen, Santa Clara, CA, United States) and qPCR (QuantStudio 5 Real-Time PCR System, Applied Biosystems, Thermo Scientific, Paisley, United Kingdom) were used for library quantification and quality estimation. High-quality, purified libraries were sequenced on an Illumina HiSeq 2500 (San Diego, CA, United States) with 2 × 250 cycle chemistry. Paired-end reads were de-multiplexed, truncated (to remove the adapter and primer sequences), and merged using FLASH (V1.2.7) (Magoè and Salzberg, 2011). The raw tags thus generated were filtered to obtain high-quality clean tags employing QIIME (V1.7.0) (Caporaso et al., 2010). The *de novo* chimera removal method was implemented in UCHIME (version 11) (Edgar et al., 2011). Based on their sequence similarity, pre-processed reads from all samples were pooled and clustered into operational taxonomic units (OTUs). The sequence similarity was established using the Usparse v7.0.1090 program (similarity cutoff = 0.97) and queried against the SSUrRNA database of SILVA Database (Quast et al., 2012).

### Bioinformatics and statistical analysis

Alpha- and beta-diversity indices were calculated with QIIME (Version 1.7.0) and displayed with R software (Version 2.15.3) (Caporaso et al., 2010). A pairwise comparison of alpha-diversity indices was made through the analysis of variance (ANOVA) method at a confidence interval of 95% (*p* ≤ 0.05). Cluster analysis was preceded by principal component analysis (PCA), which was applied to reduce the dimension of the original variables using the FactoMine R package and ggplot2 package in R software (Version 2.15.3). Analysis of similarity (ANOSIM) coefficients was calculated on weighted Unifrac distance. LEfSe analysis was conducted by LEfSe software (Segata et al., 2011). The *p*-value and *q*-value were calculated by the permutation test method and Benjamini and Hochberg false discovery rate method, respectively (White et al., 2009). The SPARCC correlation network analysis employed Kendall’s correlation test (threshold: 0.3, 100 permutations) (Watts et al., 2019). Functional annotation based on the 16S rRNA gene was done through Tax4Fun software on Silva taxonomies (Wemheuer et al., 2018). The microbial dysbiosis index (MD index) was calculated based on differentially abundant OTUs between H1, H2, and G2 (total abundance of OTUs increased in site 1) and the total abundance of OTUs decreased in site 2 (Soares et al., 2016).

## Results

In the current investigation, the bacterial and fungal populations present within the indoor aerosols of two major hospitals in Kuwait were mapped. Targetted amplicon sequencing was performed to achieve this. High-quality libraries of 13 and 16 samples for bacteria and fungi were obtained. Libraries with compromised quality (*n* = 3; two from the H1 group and one from the G2 group) were omitted for further processing. Paired-end sequencing of the 16S rRNA and ITS regions generated approximately 21 million bacterial (639,858,259 bp) and 23 million fungal (646,493,926 bp) reads. More than 94% of bases had a Q score > 30 (Supplementary Tables 1, 2). The effective% ranged between 62–82% and 53–97% for bacterial and fungal sequences, respectively. Data rarefaction was performed to check the normality of sequenced reads. All the bacterial and fungal sequences plateaued at *ca.* 10,000 bp, and therefore no exclusions were made during data analysis (Supplementary Figures 1, 2). The filtered bacterial and fungal reads were processed to obtain tags that eventually yielded the OTUs (Supplementary Figures 3, 4). The data set was further explored for taxonomies, spatial variations, alpha–beta diversity analysis, network analysis, and functional prediction.

### Taxonomic composition of bioaerosols

The baseline taxonomic composition of the bioaerosols was examined to identify the predominant forms. Considering the long list of taxa in the descending order of abundance, only the details of the first 10 taxa are presented in more detail. The complete classification of each OTU is provided in Supplementary Tables 3, 4.

**FIGURE 1:**
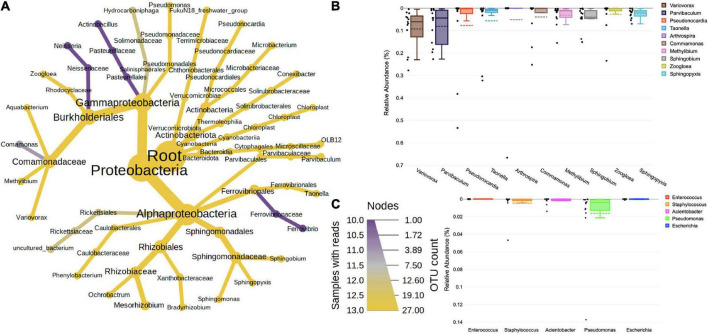
**(A)** Taxonomic composition of bacteria in bioaerosols. The Wilcoxon rank test (*p* < 0.05) was applied to median abundances of bacterial OTUs to create the differential abundance tree. The nodes represent the bacterial taxon. The size of the node denoted the OTU counts, and the color represents its presence in a number of samples. A key to the OTU count and sample prevalence is provided in the right-hand side corner. Box plots showing RA of **(B)** top 10 dominant bacterial genera and **(C)** ESKAPE pathogens. The RA are plotted on the Y-axis and the corresponding genera on the X-axis. Each box represents the inter-quartile range (25–75%), upper and lower whiskers (10–90%), and dashed lines are the mean RA%. Black dots represent the RA of the individual sample.

Majority of the 16S sequences aligned with the (Figure 1A) phyla Proteobacteria (72.54%), followed by Actinobacteria (11.39%), Cyanobacteria (6.35%), Verrumicrobiota (4.19%), Firmicutes (2.19%), and Bacteroidota (2.09%). The classes of these phyla were represented by Alphaproteobacteria (38.66%) > Gammaproteobacteria (33.88%) > Actinobacteria (8.6%) > Cyanobacteriia (6.35%) > Verrumicrobiae (4.19%) > Thermoleophilia (2.75) > Bacteroidia (2.09%) > Bacilli (1.57%) > Clostridia (0.6%) and Phycisphaerae (0.33%). The classes were further classified into orders, such as Burkholderiales (26.44%), Sphingomonadales (8.48%), Parvibaculales (8.33%), Pseudonocardiales (6.48%), Ferrovibrionales (5.23%), Chthoniobacterales (4.16%), Defluviicoccales (3.84%), Reyranellales (3.75%), and Cyanobacteriales (3.56%). The most prevalent families were Commmonadaceae (20.59%), Sphingomonadacae (8.48%), Parvibaculaceae (8.33%), Pseudomonadaceae (6.48%), Terrimicrobiaceae (4.16%), Reyranellaceae (3.75%), Phormidiaceae (3.56%), Xanthobacteraceae (2.96%), Solirubrobacteraceae (2.68%), and Rhodocyclaceae (2.47%). At the genus level (Figure 1B), *Variovorax* (9.44%), *Parvibaculum* (8.27%), *Pseudonocardia* (8.04%), *Taonella* (5.74%), *Arthrospira* (4.58%), *Comamonas* (3.84%), *Methylibium* (3.13%), *Sphingobium* (4.46%), *Zoogloea* (2.20%), and *Sphingopyxis* (2.56%) were among the highly abundant ones. Besides these, the known pathogenic forms abbreviated as ESKAPEE (*Enterococcus faecium*, *Staphylococcus aureus*, *Klebsiella pneumoniae*, *Acinetobacter baumannii*, *Pseudomonas aeruginosa*, *Enterobacter* spp., and *Escherichia coli*) were also looked at. Among these, *Pseudomonas* (0.048), *Staphylococcus* (0.049), *Acinetobacter* (0.017), and *Escherichia* (0.002) were found in low abundance (Figure 1C).

**FIGURE 2:**
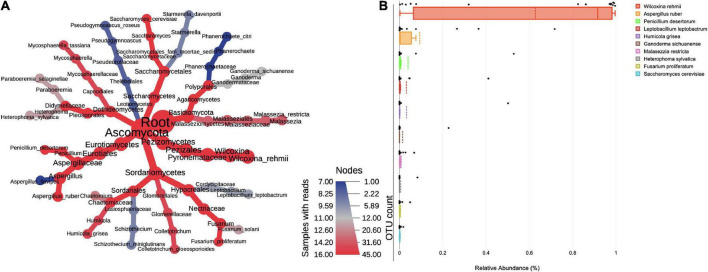
**(A)** Taxonomic composition of fungi in bioaerosols. The Wilcoxon rank test (*p* < 0.05) was applied to median abundances of fungal OTUs to create the differential abundance tree. The nodes represent the fungal taxon. The size of the node denoted the OTU counts and the color represents its presence in a number of samples. A key to the OTU count and sample prevalence is provided in the right-hand side corner. **(B)** Box plots showing RA of top 10 dominant fungal species. The RA values are plotted on the Y-axis and the corresponding genera on the X-axis. Each box represents the inter-quartile range (25–75%), upper and lower whiskers (10–90%), and dashed lines are the mean RA%. Black dots represent the RA of the individual sample.

The ITS sequencing analysis provided information on diverse fungal taxa. The entire fungal classification up to species level was presented on a heated tree (Figure 2A). The fungal phyla Ascomycota (88%) was the most dominant in bioaerosols and was followed by Basidiomycota (2.72%). The leading classes of fungi were Pezizomycetes (63.98%), Eurotiomycetes (14.95%), Sordariomycetes (7.69%), Agaricomycetes (1.79%), Malasseziomycetes (0.94%), Dothideomycetes (0.78%), Saccharomycetes (0.38%), Letiomycetes (0.20%), Mucoromycetes (0.01%), and Mortierellomycetes (0.01%). Further classification at order level revealed the presence of Pezizales (63.98%), Eurotiales (14.83%), Hypocreales (4.07%), Sordariales (3.04%), Polyporales (1.65%), Malasseziales (0.94%), Pleosporales (0.55%), Saccharomycetales (0.38%), Glomerellales (0.37%), and Capnodiales (0.23%). Orders were distributed into families, namely Pyronemataceae (62.87%), Aspergillaceae (14.83%), Cordycipitaceae (2.85%), Chaetomiaceae (2.82%), Ganodermataceae (1.27%), Necteriaceae (1.17%), Malasseziaceae (0.94%), Didymellaceae (0.52%), Phanerochaetaceae (0.38%), and Glomerellaceae (0.31%). The predominant genera and their species were *Wilcoxinia rehmii* (64.38%), *Aspergillus ruber* (9.11%), *Penicillium desertorum* (3.89%), *Leptobacillium leptobactrum* (3.20%), *Humicola grisea* (2.99%), *Ganoderma sichuanense* (1.42%), *Malassezia restricta* (0.74%), *Heterophoma sylvatica* (0.49%), *Fusarium proliferatum* (0.46%), and *Saccharomyces cerevisiae* (0.23%) (Figure 2B). The species of *Penicillium*, *Aspergillus*, *Fusarium*, and *Saccharomyces* cause diseases in humans, plants, and animals.

### Spatial variations in bacterial and fungal communities

**FIGURE 3:**
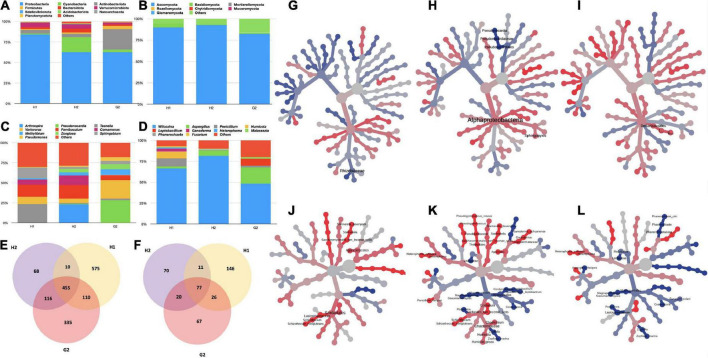
Variation in RA% of bacterial and fungal taxa in hospitalized (H1 and H2), and non-hospitalized (G2) settings. **(A)** Variations in bacterial phyla; **(B)** variations in fungal phyla; **(C)** variations in bacterial genera; and **(D)** variations in fungal genera. For all the bar plots, the groups are plotted on the x-axis, and relative abundances (RA) are shown on the y-axis. The color key to each taxon is presented on the top of each bar graph. OTU Clustering analysis: **(E)** Common and unique bacterial OTUs; **(F)** common and unique fungal OTUs. Differential abundance analysis **(G)** between bacterial taxa of H1 and H2; **(H)** between bacterial taxa of H1 and G2; **(I)** between bacterial taxa of H2 and G2; **(J)** between fungal taxa of H1 and H2; **(K)** between fungal taxa of H1 and G2; and **(L)** between fungal taxa of H2 and G2. The Wilcoxon rank test (*p* < 0.05) was applied to median abundances of OTUs to create the differential abundance tree.

The infrastructure and geographic location of each hospital differed, and the likelihood of microbes unique to each site was much expected. To prove this, a comparison of the RA of prevalent microbes in two hospitals (H1 and H2) and the non-hospital (G2) site was performed in the first place (Figure 3A). In bacteria, the phylum Proteobacteria was highest in abundance at H1, H2, and G2. Maximum RA (83.0%) was recorded at H1, followed by H2 and G2 (*ca.* 62.0%). The second in the list at H1 was phylum Verrumicrobiota (5.3%), followed by Actinobacteria (4.3%) > Bacteriodota (1.9%) > Cyanobacteria (1.3%) > Firmicutes (1.2%). H2 followed a similar trend to H1. The order of abundance at G2 differed with Actninobacterota (24.6%) > Firmicutes (3.9%) > Cyanobacteria (3.0%), Verrumicrobiota (2.4%). The fungal phyla varied in their RA, but their order of dominance was almost equal in all the sites. The RA of Ascomycota (Figure 3B) was strikingly higher at H1 and H2 (*ca.* 90%). At G2, it was 82.0%. The next phylum in the order of abundance at all the locations was Basidiomycota (H1: 4.65%, H2: 0.4%, and G2: 2.0%). This was followed by Mortierellomycota (<0.001%). Variations in RA at the lower taxonomic levels of class, order, family, genus, and species were also recorded. The description of bacterial genera and fungal species specific to each hospital is described in the following text.

Among the bacterial genera (Figure 3C), at H1 *Taonella* (15.4%) was maximum, followed by *Parvibaculum* (9.6%), *Sphingobium* (8.5%), *Variovorax* (5.9%), *Comamonas* (4.3%), *Methylibium* (1.2%), *Pseudomonas* (0.43%), *Zoogloea* (0.16%), *Pseudonocardia* (0.11%), and *Arthrospira* (0.0%). At H2, *Arthrospira* (13.9%) was the highest in abundance with *Parvibaculum* (10.3%) at second place, followed by *Comamonas* (7.1%), *Variovorax* (3.2%), *Methylibium* (2.2%), *Zoogloea* (2.5%), *Sphingobium* (1.5%), and *Pseudomonas* (1.0%). Unlike the hospital locations, at G2, *Pseudonocardia* (19.1%) was highest, followed by *Variovorax* (15.6%), *Methylibium* (4.8%), *Parvibaculum* (4.2%), *Pseudomonas* (2.7%), *Sphingobium* (0.27%), and *Taonella* (1.2%) in the order of their RA. The fungal genera also varied in RA at all three sites (Figure 3D). The genus *Wilcoxinia* was highest at all three sites (H1: 64.3%, H2: 81.0%, and G2: 47.5%). The second-most abundant fungal genera at H1 were *Penicillium* (9.4%), followed by *Humicola* (7.6%), *Ganoderma* (3.65), and *Heterophoma* (1.2%). At H2, *Aspergillus* was the second-highest level and was followed by *Penicillium.* The G2 had *Aspergillus* (19.0%) and *Leptobacillium* (8.1%) in second and third places, respectively.

It was important to see the variations in the abundance of ESKAPEE genera, as potential outbreaks caused by some of their species may cause serious consequences. The RA of ESKAPE genera also varied according to the sampling location. *Enterococcus* was only detected at H1. *Acinetobacter* exhibited a maximum RA of 2.1% at G2 and 0.2% at H1 and H2. The genus *Pseudomonas* (15.8%) was maximum at G2. Its abundance at H1 and H2 was 1.7 and 3.7%, respectively. *Staphylococcus* was almost comparable at H1 and H2, whereas its abundance was recorded as 5.3% at G2 (Table 2). Genus *Escherichia* was recorded both at H1 and H2; however, at G2 it was negligible. Fungal species of *W. rehmii* were recorded at H1 (66.4%), H2 (82%), and G2 (48.8%). A noticeable abundance of *A. ruber* (18%) was recorded at G2 and H2 (6.7%). At H1, *P. desertorum* (8.9%) was the next in abundance after *W. rehmii* (Table 2).

**TABLE 2:** Spatial variations in relative abundance (%) of ESKAPE pathogens and fungal species.

Taxa	Sampling location (Relative abundance %)		
	
	H1	G2	H2
**Bacteria**			
*Enterococcus*	0.001	0.000	0.000
*Acinetobacter*	0.002	0.021	0.002
*Staphylococcus*	0.004	0.053	0.002
*Pseudomonas*	0.017	0.158	0.037
*Escherichia*	0.001	0.002	0.000
**Fungi**			
*Wilcoxina rehmii*	0.664	0.488	0.820
*Penicillium desertorum*	0.089	0.014	0.001
*Humicola grisea*	0.079	0.000	0.001
*Ganoderma sichuanense*	0.037	0.000	0.000
*Aspergillus ruber*	0.019	0.177	0.067
*Heterophoma sylvatica*	0.013	0.000	0.000
*Fusarium proliferatum*	0.008	0.002	0.003
*Malassezia restricta*	0.005	0.014	0.002
*Saccharomyces cerevisiae*	0.004	0.001	0.000

Additional analyses were performed to compare the inter-site variability among the bacterial and fungal taxa through OTU clustering. The common and unique features were recorded at each sampling site. We discovered 455 common OTUs (bacterial) in the aerosols and 575, 68, and 335 OTUs unique to H1, H2, and G2, respectively (Figure 3E). There was 10 OTUs common between H1 and H2, 110 were common between H1 and G2, and 116 were common between H2 and G2. In the case of fungi, 146, 67, and 70 OTUs were unique to H1, H2, and G2 respectively. Seventy-seven were common between all the sites, with 11 common between H1 and H2, 26 common between H1 and G2, and 20 common between G2 and H2 (Figure 3F).

Pairwise comparison of differential abundance through Wilcoxson’s sum test (*p* > 0.05) also returned significantly variant taxa among the three sites. In the bacterial category, the family Rhizobiaceae differed between H1 and H2 (Figure 3G). Similarly, class Alphaproteobacteria, order Pseudonocardiales, family Pseudonocardiaceae, and genera *Pseudonocardia* and *Sphingopyxis* differed between H1 and G2 (Figure 3H). The single-order Defluvicoccales varied significantly between H1 and G2 (Figure 3I). The fungal taxa exhibited more variations between the three sites (Figures 3J–L). A total of 8, 35, and 17 taxa were significantly differentially abundant among H1-H2, H1-G2, and H2-G2 groups, respectively.

### Intra-site variability

**FIGURE 4:**
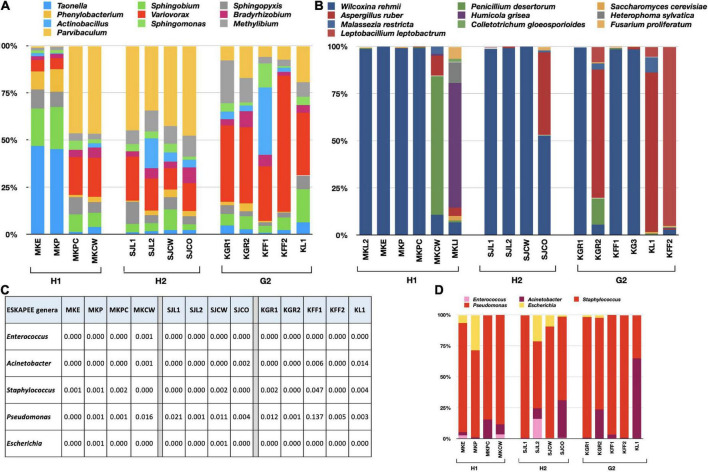
Bar charts showing the variations of RA within each sub-location of H1, H2, and G2. **(A)** Bacterial genera, **(B)** fungal species, **(C)** RA of ESKAPEE genera, and **(D)** bar chart showing variations in RA of ESKAPEE genera. The RA is plotted on the y-axis and the corresponding taxa on the x-axis.

The RA of bacterial genera varied within the sub-locations of H1, H2, and G2 (Supplementary Table 5). 16S rRNA results were obtained for four locations within H1 and H2, and five in G2. The top 10 genera were compared for variations in their RA within each site (Figure 4A). At H1, *Taonella* was most abundant in MKE (area near the main entrance) and MKP (reception of the main pharmacy). The RA of *Sphingobium* was also high when compared to other genera found at MKE and MKP. The genus *Parvibaculum* was maximum in abundance at MKPC (pediatric causality ward) and MKCW (COVID-19 isolation ward). The second-most abundant genera in the same places were *Variovorax.* At H2, genus *Parvibaculum* was highest at SJL1 (cytology laboratory), SJL2 (virology laboratory), SJCW (COVID ward), and SJCO (COVID-observation ward). The next dominant genus at all these sub-locations in H2 was *Variovorax.* In the case of G2, *Variovorax* was more commonly found in KGR1 (main reception), KGR2 (reception near the canteen), KFF1 (corridor on the first floor near the lift), KFF2 (corridor near the staircase and electrical unit), and KL1 (the area inside the COVID research laboratory). *Actinobacillus* was high at KFF1. In general, it was observed that the G2 bacterial genera exhibited more variations when compared to H1 and H2. This could be attributed to the similar air-conditioning, ventilation system, and sanitization followed in the hospitals when compared to G2.

The ITS sequences of six sub-locations of H1 and G2, and four sub-locations of H2 also exhibited variations in RA of fungal species (Supplementary Table 6). *W. rehmii* dominated at MKL2, MKE, MKP, and MKPC, whereas *P. desertorum* and *H. grisea* were overwhelmingly higher at MKCW and MKL1 (Figure 4B). The remaining species exhibited very low abundances. At H2, except at SJCO (maximum prevalence of *A. ruber*), again *W. rehmii* was highest at SJL1, SJL2, and SJCW. At G2, either *W. rehmii* (KGR1, KFF1, and KG3) or *A. ruber* (KGR2, KL1, and KFF2) were among the highly prevailing fungal species. These results were in congruence with the findings of the alpha-diversity analysis.

Variations in ESKAPEE genera were also looked at carefully, and similar patterns in RA abundances were recorded (Figure 4C). *Enterococcus* was sparingly present at MKCW and MKE, and was absent from all other locations of H1. At H2, its presence was only detected at SJL2. It was not detected at all at G2. In the case of *Acinetobacter*, exceptionally high abundances were recorded at KL1 (G2), whereas RA > 0.001 was recorded at other sub-locations of G2, H2, and H1. *Staphylococcus* was more at MKE, MKP, and MKPC within H1. Its abundance was comparable at MKCW, SJL1, SJL2, SJCO, SJCW, KGR1, KGR2, KFF1, KFF2, and KL1. *Pseudomonas* species were overwhelmingly high among the ESKAPEE genera. Its presence was recorded within all the sub-locations of H1, H2, and G2. On average, it was more at G2 (KGR1, KGR2, KFF1, KFF2, and KL1). *Escherichia* was only found at MKE and MKP within H1; SJL2, SJL2, SJCW, and SJCO within H2; and at KGR1 and KGR2 within G2 (Figure 4D). Pairwise comparisons through student’s *t*-test revealed *p*-values above 0.05, rendering the variations insignificant.

### Alpha- and beta-diversity analysis

**FIGURE 5:**
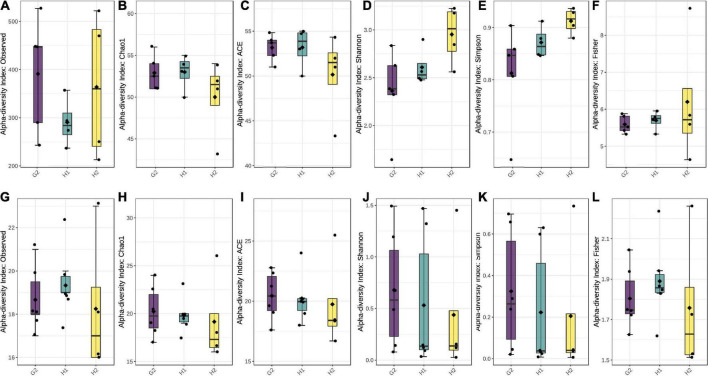
Box plots with alpha-diversity indices: **(A–F)** for bacterial species and **(G–L)** for fungal species. The boxes represent the range of alpha diversity within each location. Corresponding alpha-diversity indices (observed, Ace, Chao1, Shannon’s, Simpson, and Fisher) are plotted on Y-axis. All the comparisons were done employing Tukey’s and Wilcox’s tests at *p* ≤ 0.05.

Each hospital had a unique infrastructure, most likely contributing to differences within and among the sites. To confirm this, the alpha- and beta-diversity analyses were performed. Intra-site diversity was assessed using alpha-diversity indices of observed species, ACE, Chao1, Shannon’s index, Simpsons, and Fisher index. Over 99% of all the samples had adequate biodiversity captured at each site (Supplementary Tables 7, 8). Data rarefaction was also performed with a plateau obtained at 10,000 sequences for bacteria (Supplementary Figure 3) and fungi (Supplementary Figure 4), indicating that sufficient diversity was captured through sequencing. For bacterial species, the highest diversity was at G2, followed by H2 and H1 (Figure 5A). Chao1 and ACE were comparable at G2 and H1 with slightly lower values at H2 (Figures 5B,C). The Shannon and Simpson were highest at H2, with the second-highest numbers at H1 and H2 (Figures 5D,E).

Fisher values were almost comparable at all three locations (Figure 5F). A comparison of all the alpha-diversity indices by Tukey’s and Wilcox test revealed *p*-values > 0.05, indicating that within-site diversity was not significant. This could be attributed to the similar interior settings within each sampling location.

In the case of fungi, all the alpha-diversity indices, except for observed and Fisher, were highest at G2, followed by H1 and H2 (Figures 5G–L). The observed species were highest at G2 and lowest at H2. Likewise, Fischer indices were highest at H1 and second-highest at G2, followed by H2 (Figures 5J–L). This indicates both species richness and evenness of the fungal population to be higher in ambient air than in indoor aerosol. Among the indoor site, the non-hospital settings (G2) had comparatively higher fungal counts than hospital settings (G1). Unlike the bacterial population, the fungal alpha-diversity indices of observed, Chao1, and Fischer were significant at *p* < 0.05 compared with Tukey’s and Wilcox test.

**FIGURE 6:**
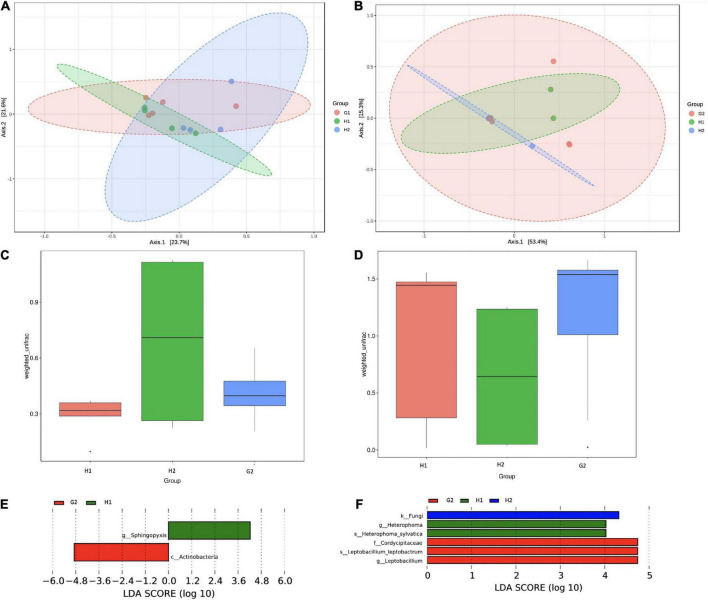
Beta-diversity analysis of microbial communities of indoor aerosols of hospitalized and non-hospitalized settings in Kuwait. **(A)** PCA plot of bacterial communities, **(B)** PCA plot of fungal communities, **(C)** ANOSIM analysis of bacterial community, **(D)** ANOSIM analysis of fungal communities, **(E)** LDA analysis of bacterial taxa, and **(F)** LDA analysis of fungal taxa. LDA scores are plotted on the x-axis on a logarithmic scale. The corresponding taxa are shown on the Y-axis. The LEfSe algorithm applying the non-parametric factorial Kruskal–Wallis (KW) sum-rank at an FDR (q) ≤ 0.05 followed by the linear discriminant analysis (LDA) was employed to select significantly differential taxa.

The differences in community structure were analyzed through the beta-diversity analysis. The clustering of Bray-Curtis distances of bacterial communities distributed the samples into three overlapping groups. As evident from Figure 6A, the H1 and H2 communities lay close, whereas the G2 was slightly apart. Permutational multivariate analysis of variance (Adonis) was performed on UniFrac; distances between H1-H2-G2 (indoor aerosols of hospitalized and non-hospitalized settings) showed the difference in the community to be moderate but significantly diverse (*r*^2^ = 0.117; *p* = 0.046) (Figure 6C). The PCA analysis showed the fungal communities at H1 and H2 to be closer but distantly apart from G2 (Figure 6B). The Adonis coefficients were 0.069 and demonstrated insignificant *p*-values (*p* = 0.263) (Figure 6D). Pairwise Unifrac distances between samples are provided in Supplementary Figures 5, 6.

The linear discriminant analysis was performed to identify key indicator taxa between H1-H2-G2. The LefSe algorithm identified two bacterial taxa to be significantly abundant (FDR q ≤ 0.05) at H1 (order Defluvicoccales) and G2 (class Actinobacteria) (Figure 6E). Within the fungal communities, the differentially abundant taxa were kingdom fungi at H2, genus *Heterophoma*, and species *H. sylvatica* at H1, and family Cordycipitaceae, genus *Leptobacillium*, and species *L. leptobactrum* at G2 (Figure 6F). The observations were in partial agreement with the differential abundance testing.

### Network analysis and functional prediction

**FIGURE 7:**
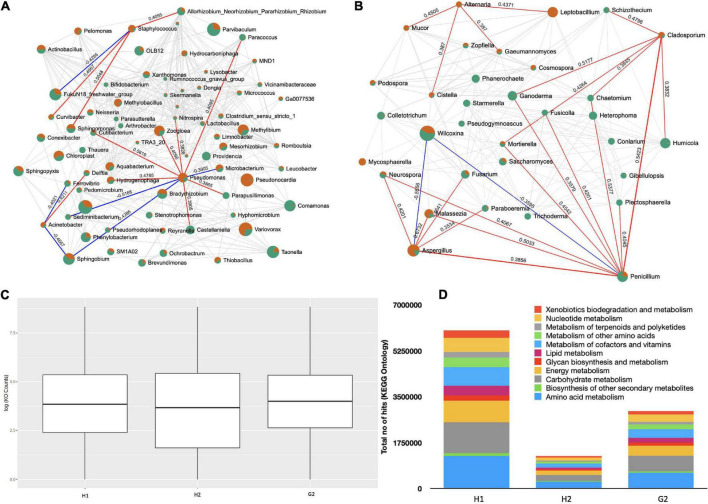
Network analysis and functional prediction: **(A)** Network lattice of bacterial genera, **(B)** network lattice of fungal species, **(C)** functional prediction of bacterial 16s rRNA genes, and **(D)** major KEGG pathways in bacterial genera.

The Kendall correlation among the top 100 genera was investigated. The network analysis (100 permutations; *p* < 0.05) returned complex lattices showing positive and negative relationships. The interactions were more complex among bacterial genera (Figure 7A) represented by 399 edges (an average of 5.55 edges per node). Of these, 52 were negative and 347 were positive. Interestingly, three of the ESKAPE genera were part of the network. *Pseudomonas* was positively correlated with seven genera (*Zoogloea:* 0.409, *p* = 0.033; *Castellaniella*: 0.390, *p* = 0.042; *Cutibacterium*: 0.561, *p* = 0.003; *Hydrogenophaga*: 0.478, *p* = 0.013; *Nitrospira*: 0.382, *p* = 0.047; *Paracoccus*: 0.409, *p* = 0.033; and *Parapusillimonas*: 0.386, *p* = 0.046) and negatively with three genera (*Sediminibacterium*: 0.516, *p* = –0.007; *Microbacterium*: 0.418, *p* = –0.0381; and *Sphingobium*: 0.4286, *p* = –0.026). Similarly, *Acinetobacter* was positively correlated with *Delftia* (0.421; *p* = 0.029) and negatively with *Ferrovibrio* (−0.402; *p* = 0.040) as well as *Sphingobium* (−0.466; *p* = 0.015). The genus *Staphylococcus* correlated positively with *Allorhizobium* (0.409; *p* = 0.033), *Curvibacter* (0.466; *p* = 0.015), and *Sphingomonas* (0.504; *p* = 0.008). It interacted negatively with a marine bacterium (−0.428, *p* = 0.026). To see the shifts in microbial communities toward diseased states in the indoor environments of hospitals, the microbial dysbiosis (MD) index was estimated. The H1/H2, H1/G2, and H2/G2 MD index was −0.6522, −0.2148, and 0.9305, respectively.

The fungal genera were divided into 187 correlations (5.55 edges per genera) (Figure 7B). In total, 155 were positive and 32 negative. Many of the fungal forms were pathogenic; however, we only looked at the correlations that were most common, such as *Alternaria*, *Aspergillus*, *Penicillium*, and *Fusarium*. *Alternaria* interacted positively with *Cistella* (0.387, *p* = 0.046), *Gaeumannomyces* (0.387, *p* = 0.046), *Leptobacillium* (0.437, *p* = 0.018), and *Mucor* (0.450, *p* = 0.019). *Aspergillus* made positive associations with *Fusarium* (0.464, *p* = 0.007), *Malassezia* (0.673, *p* = 0.000), *Neurospora* (0.420, *p* = 0.016), *Paraboeremia* (0.353, *p* = 0.043), and *Penicillium* (0.385, *p* = 0.025). *Penicillium* was in positive correlation with *Plectosphaerella* (0.405, *p* = 0.029), *Aspergillus* (0.385, *p* = 0.025), *Chaetomium* (0.537, *p* = 0.001), *Cladosporium* (0.542, *p* = 0.002), *Fusicolla* (0.420, *p* = 0.024), *Malassezia* (0.503, *p* = 0.035), *Mortierella* (0.454, *p* = 0.010), *Neurospora* (0.406, *p* = 0.020), and *Ganoderma* (0.357, *p* = 0.044). *Fusarium* exhibited positive correlations with *Gaeumannomyces* (0.506, *p* = 0.007), *Malassezia* (0.426, *p* = 0.013), *Mortierella* (0.371, *p* = 0.036), *Mucor* (0.482, *p* = 0.009), *Mycosphaerella* (0.406, *p* = 0.018), *Paraboeremia* (0.553, *p* = 0.001), *Phanerochaete* (0.637, *p* = 0.000), *Podospora* (0.490, *p* = 0.009), *Cosmpospora* (0.522, *p* = 0.005), *Aspergillus* (0.464, *p* = 0.007), *Cistella* (0.539, *p* = 0.004), and *Colletotrichum* (0.411, *p* = 0.017). There were more opportunistic invaders in the fungal network when compared to bacteria and therefore comparatively higher H1/H2, H1/G2, and H2/G2 microbial dysbiosis (MD) index of −1.227, 3.062, and 2.34 were recorded, respectively.

A functional profile of bacterial communities was generated using the Tax4Fun to understand their metabolic contribution to the aerosol ecosystem. The OTUs mapped against 6,134 Kyoto Encyclopedia of Genes and Genomes (KEGG) terms. The log 10 counts of KEGG ontologies were at comparable levels at H1, H2, and G2 (Figure 7C). The KEGG pathways were part of 11 major metabolic pathways as shown in Figure 7D. The majority of them belonged to amino acid metabolism, biosynthesis of secondary metabolites, carbohydrate, energy, glucan, and lipid metabolisms, to name a few. Further details are provided in Supplementary Table 9. The absolute counts of KO terms of these functions were in the order of H1 > H2 > G2.

## Discussion

Nosocomial aerosols are carriers of diverse microbial communities, including bacteria, fungi, and viruses. Inhalation of these microbes poses a 2.5 times higher risk to the hospital staff and frequent hospital visitors (Macintyre et al., 2012). Hospital-mediated outbreaks have resulted in severe infections (Yang et al., 2013; O’connor et al., 2015; Park et al., 2018; Weber et al., 2019; Løvestad et al., 2021; Paltansing et al., 2021), making the bioaerosol assessment in indoor hospital environment highly desirable. Earlier studies reporting both the bacteria and fungi simultaneously in the built environments of hospitals have relied on the traditional culture-dependent methods (Sarıca et al., 2002; Ekhaise et al., 2010; Okten and Asan, 2012; Sudharsanam et al., 2012; Park et al., 2013). The NGS approach for microbial community characterization has been a method of choice in the last few years owing to its fast, accurate, and precise estimation (King et al., 2016; Tong et al., 2017; Gao et al., 2018; Comar et al., 2019; Chakrawarti et al., 2020).

In this study, the bacterial and fungal communities were identified using the NGS approach at the two hospitals (H1 and H2) and non-hospital settings (G2). The community composition analysis of hospital indoor aerosols revealed phyla Proteobacteria was dominant among bacteria and Ascomycota among the fungi. Similar trends are reported elsewhere; the highest abundances of Proteobacteria were recorded in aerosols collected from the Delhi University Health Center, India (Chakrawarti et al., 2020), and urban hospitals in China (Gao et al., 2018). Ascomycota was found in four departments of a hospital in Beijing (Tong et al., 2017). Exhaled air seems to be a significant source of these communities within the indoor aerosols of hospitals (Prussin and Marr, 2015). This observation is further supported by a study conducted at the University of Chicago Medical Center that reports microbiota on surfaces within a patient room resembled the patient microbial community (Lax et al., 2017). Several other studies reported taxa from indoor air commonly found in human orifices and respiratory tract (Charlson et al., 2011; Nakatsuji et al., 2013; Madi et al., 2018; Habibi et al., 2021b). A study very well-demonstrated the microbiome of a hospital became dominated by a human skin-associated microbe as soon as it became operational (Lax et al., 2017).

Several studies have revealed the presence of different bacterial genera. For instance, a longitudinal metagenomic analysis of hospital air identified *Stenotrophomonas* as an opportunistic invader (King et al., 2016). In a university health center in Delhi, *Psychrobacter*, *Arthrobacter*, and the ESKAPE (*Enterococcus faecium*, *Staphylococcus aureus*, *Klebsiella pneumoniae*, *Acinetobacter baumannii*, *Pseudomonas aeruginosa*, and *Enterobacter* spp.) pathogens were recorded (Chakrawarti et al., 2020). *Acinetobacter*, *Enterobacter*, *Pseudomonas*, and *Staphylococcus* were the common genera identified in four healthcare institutes in Taiwan (Chen et al., 2017). Unlike the above studies, we recorded the presence of *Variovorax*, *Parvibaculum*, *Pseudonocardia*, *Taonella*, *Methylibium*, *Sphingobium*, *Arthrospira*, *Comamonas*, *Sphingopyxis*, and *Zoogloea*. Five of the ESKAPEE with very low RA pathogens were also discovered along with these predominant forms. Due to differences in sample methodologies, laboratory protocols, bacterial primer selection, sequencing methods, and data analysis pipelines, cross-study comparisons of bacterial and fungal genera are unequivocal. Similarly, the fungi observed were *Wilcoxinia*, *Penicillium*, *Aspergillus*, *Humicola*, *Ganoderma*, *Heterophoma*, *Leptobacillus*, *Malassezzia*, *Saccharomyces*, and *Fusarium.* Among these, *Penicillium* and *Aspergillus* were also found in the clinical microbiology laboratory of a university teaching hospital (Nagano et al., 2009). High-throughput sequencing revealed the prevalence of *Aspergillus* in the Respiratory Intensive Care Unit, Intensive Care Unit, Emergency Room, and Outpatient Department of a general hospital in Beijing (Tong et al., 2017).

From a health perspective, species identification is recommended as the bacterial and fungal species interact with the host to establish an antagonist, symbiotic, or synergistic relationship. However, due to the technical limitation of the 16S rRNA gene sequencing, we cannot go for the species-level classification for bacterial forms. A shotgun metagenomic approach will be followed in the future to gain knowledge of the bacterial species. Nevertheless, the presence of ESKAPEE genera (RA < 0.01) in our samples cannot be ignored. Analogous to our findings, unclassified species of *Pseudomonas*, *Acinetobacter*, and *Staphylococcus* were reported in extremely low percentage in aerosols of university healthcare center in Delhi (Chakrawarti et al., 2020). The concern also exuberates from the presence of some opportunistic pathogenic fungi, such as *Aspergillus ruber* (Lewis et al., 1994) (Guo et al., 2021), *Colletrotrichum gloeosporiodes* (Lin et al., 2015), *Malassezia restricta* (Houhamdi-Hammou et al., 2021), *Fusarium proliferatum* (Seyfarth et al., 2008), and *Penicilium desertorum* (Durmaz et al., 2021) in indoor hospital air. Besides these, the species of *Wilcoxinia rehmii* (Fenn et al., 2011), *Ganoderma sichuaense* (Xinsheng et al., 1995), and *Humicola grisea* (Andrioli et al., 2008) are plant pathogens and mycotoxin releasing forms. These might be considered major role players in causing allergic responses upon inhalation. It is, therefore, prudent to further investigate the infectivity dose of the pathogenic microbes upon inhalation.

Variations in alpha-diversity indices of bacterial and fungal communities were observed, although pairwise comparisons were non-significant. A small sample size most likely contributes to a higher p-value in our case. A substantial proportion of other genera with very low RA probably explains the variations in species richness and evenness (Lax et al., 2017) in the present samples. We would also like to mention that a reasonable number of unique OTUs were recorded at each sampling site (Rampelotto et al., 2019). Sogin et al. (2006) termed these features with low abundance as “rare biosphere” and proposed them to be representing some hidden functions. Also, the species abundance and richness were higher in the ambient air samples than in the indoor samples of hospital and non-hospital settings. This is attributed to the strict cleanliness and disinfection procedures followed at the Kuwaiti hospital consequent of the ongoing SARS-CoV-2 pandemic (Habibi et al., 2021d). A recent review published that about 80% of studies reported a decrease in hospital-associated infectious microbes upon following intensive healthcare environmental hygiene (Peters et al., 2022). The LEfSe analysis revealed the key indicator taxa as Defluvicoccales, *Heterophoma*, and *H. sylvatica* in hospitals when compared to Actinobacteria, *Leptobacillium*, *L. leptobacillium*, and Cordycipitaceae at G2 (KISR), all with very low RA.

We supposed the microbial community structure to widely differ between the hospital and non-hospital sites. We recorded a moderately diverse community structure of the bacterial population (ANOSIM *r*^2^ = 0.181–0.243; *p* < 0.05), whereas the fungal population was more or less homogenous at all the sampling locations. The small difference in bacterial community is due to the change in their RA (Jiayu et al., 2019). In congruence with our study, the assessment of bioaerosol particles from hospital wards and operating theaters in Tehran revealed slightly higher diversity in bacterial communities when compared to the fungal population (Bolookat et al., 2018). Moderately diverse bacterial communities were also observed in the indoor atmosphere of a Brazilian hospital (Rampelotto et al., 2019). Contrary to this, high fungal diversity was recorded in the indoor environments of Beijing hospital (Tong et al., 2017). Community structure is defined by the socio-economic status, human footfall, and the general architecture of the built environment (Kembel et al., 2012). All the buildings in Kuwait are centrally air-conditioned with controlled ventilation (ANON, 2008). This also accounts for the non-significant intra-site variations observed in H1, H2, and G2. Besides these, the non-hospital indoor site is an institute that is actively involved in COVID-19 research during the entire sampling period (Habibi et al., 2021a,d,g,2022b), and is accompanied by frequent visits by the staff members to these hospitals for sample collection, COVID-19 checkups, vaccination campaigns, etc. Hence, a more or less similar community structure between both the indoor sites is well-justified.

The community structure of bioaerosols plays an important role in public health, as a single pathogen cannot determine whether a diseased condition will be established (Jiayu et al., 2019). The network analysis revealed their involvement with each other and strengthens our belief that it is a complete microbial consortium that plays a role in dysbiosis. Rampelotto et al. (2019) suggested the positive correlations to be synergistic, while the negative correlations manifested antagonist interaction. The MD index > 2.0 suggested enrichment of fungal communities in hospital aerosols, respectively (Soares et al., 2016). In addition to this, we also found the association of these microbes (Bacteria) with several metabolic pathways. In partial agreement with our results, the functional profiling identified the metabolic pathways, such as citrate cycle (TCA), signal transduction mechanisms, bisphenol degradation, tyrosine metabolism, transcription factors, and antibiotic resistance in bacteria present on the environmental surfaces of a public hospital in South Africa (Shobo et al., 2020). Significant involvement of pathways in human diseases was also recorded by the same group. The biodegradation and metabolism of xenobiotics were one of the dominant pathways recorded in the present samples. The occurrence of antibiotic resistance genes in the microbes residing in the hospital environments is known (He et al., 2020; Li et al., 2021; Zhou et al., 2021), creating further need to understand the air resistome in the hospital indoors.

## Conclusion

The 16s RNA and ITS amplicon sequencing successfully identified the bacterial and fungal communities of bioaerosols from hospitals in Kuwait. The microbial community consisted of pathogenic genera, although at lower abundances. These microbial populations varied spatially and were enriched in the hospital environments. The bacterial population was involved in a variety of metabolic pathways, including xenobiotic degradation. The baseline information gained from this study suggests a need to design a strategy for regular monitoring of indoor bioaerosols in a hospital-built environment as an early warning system for any pathogenic outbreak. It will be interesting to consider AMRs in these bioaerosols in future studies.

## Data availability statement

The datasets presented in this study can be found in online repositories. The names of the repository/repositories and accession number(s) can be found below: NCBI SRA BioProject, accession numbers: PRJNA796511 (SRR17577942 to SRR17577943) and PRJNA796566 (SRR17579028 442 to SRR17579046).

## Author contributions

NH and SU: conceptualization and reviewing and editing. NH, SU, and FA: methodology. NH: software, data curation, original draft preparation, and visualization. NH, SU, and MB: validation. FZ, AS, FA, and NR: formal analysis. SU: investigation. SU and MB: resources. FA and SU: supervision. MB: project administration. NH and MB: funding acquisition. All authors have read and agreed to the published version of the manuscript.
