# Supplementary material for: Bacterial and fungal communities in indoor aerosols from two Kuwaiti hospitals
Source: Front Microbiol. 2022 Jul 28;13:955913. doi: 10.3389/fmicb.2022.955913 (PMC9366136; doi:10.3389/fmicb.2022.955913)
Supplement: Supplementary file 1 [file Data_Sheet_1.docx]

*Supplementary Material*

Bacterial and Fungal Communities in Indoor Aerosols from Kuwait’s Hospitals

Nazima Habibi ^1^, Saif Uddin ^1*^, Montaha Behbehani ^1^, Fadila Al Salameen^1^, Nasreem Abdul Razzack^1^, Farhana Zakir^1^, Anisha Shajan^1^, Faiz Alam^1^

^1^ Environment and Life Science Research Centre, Kuwait Institute for Scientific Research, Kuwait

***Correspondence:** Saif Uddin; sdin@kisr.edu.kw

Keywords: Bioaerosol _1_, bacteria _2_, fungi _3_, virus _4_, exhaled air _5_

**Raw Data and Quality Control**

16s rRNA and ITS amplicons were sequenced on Illumina paired-end platform to generate 250 bp paired-end raw reads (Raw PE), and then merged and pretreated to obtain Clean Tags [Caporaso et al., 2011]. The chimeric sequences in Clean Tags were detected and removed to obtain the Effective Tags which can be used for subsequent analysis. The summarizations obtained in each step of data processing are shown in table S1 (bacterial sequences) and table S2 (fungal sequences)

**Table S1: QC stats of bacterial sequences**

| Sample Name | Sub-Sample Name | Raw PE(#) | Raw Tags(#) | Clean Tags(#) | Effective Tags(#) | Base(nt) | AvgLen(nt) | Q20 | Q30 | GC% | Effective% |
| --- | --- | --- | --- | --- | --- | --- | --- | --- | --- | --- | --- |
| G2 | KFF1 | 123,929 | 98,043 | 94,808 | 82,379 | 34,963,692 | 424 | 97.76 | 93.23 | 53.97 | 66.47 |
|  | KFF2 | 126,729 | 102,328 | 100,047 | 91,222 | 37,776,752 | 414 | 97.97 | 93.76 | 57.2 | 71.98 |
|  | KGR1 | 124,751 | 96,543 | 93,926 | 76,504 | 32,038,241 | 419 | 97.98 | 93.71 | 54.68 | 61.33 |
|  | KGR2 | 132,468 | 110,947 | 108,611 | 92,547 | 38,543,471 | 416 | 98.12 | 94.07 | 55.14 | 69.86 |
|  | KL1 | 122,833 | 103,959 | 102,262 | 99,348 | 40,944,288 | 412 | 98.06 | 93.96 | 55.05 | 80.88 |
| H1 | MKCW | 133,917 | 105,880 | 103,150 | 84,954 | 35,565,838 | 419 | 98.01 | 93.82 | 54.81 | 63.44 |
|  | MKE | 131,424 | 99,932 | 98,553 | 93,043 | 37,926,490 | 408 | 98.35 | 94.57 | 54.46 | 70.8 |
|  | MKPC | 133,834 | 114,947 | 113,379 | 106,164 | 43,571,585 | 410 | 98.37 | 94.7 | 54.43 | 79.33 |
|  | MKP | 136,410 | 111,105 | 109,655 | 101,747 | 41,466,395 | 408 | 98.41 | 94.71 | 54.69 | 74.59 |
| H2 | SJCO | 135,152 | 110,409 | 108,466 | 98,971 | 40,885,215 | 413 | 98.26 | 94.36 | 54.28 | 73.23 |
|  | SJCW | 120,396 | 91,132 | 88,960 | 81,613 | 34,011,178 | 417 | 98.08 | 93.86 | 54.74 | 67.79 |
|  | SJL1 | 138,299 | 118,507 | 116,119 | 109,112 | 45,924,502 | 421 | 98.09 | 93.97 | 54.18 | 78.9 |
|  | SJL2 | 129,069 | 98,981 | 94,565 | 88,350 | 35,894,577 | 406 | 98.24 | 94.37 | 53.24 | 68.45 |

**Table S2: QC stats of fungal sequences**

| **Sample Name** | **Sample Name** | **Raw PE(#)** | **Raw Tags(#)** | **Clean Tags(#)** | **Effective Tags(#)** | **Base(nt)** | **AvgLen(nt)** | **Q20** | **Q30** | **GC%** | **Effective%** |
| --- | --- | --- | --- | --- | --- | --- | --- | --- | --- | --- | --- |
| G2 | KFF1 | 136,978 | 113,243 | 104,965 | 104,340 | 36,925,883 | 354 | 98.91 | 96.44 | 53.28 | 76.17 |
|  | KFF2 | 126,560 | 111,708 | 106,912 | 99,606 | 34,069,110 | 342 | 98.89 | 96.32 | 56.44 | 78.7 |
|  | KG3 | 121,546 | 117,615 | 116,750 | 116,336 | 40,413,287 | 347 | 99.06 | 96.85 | 52.4 | 95.71 |
|  | KGR1 | 120,831 | 114,345 | 112,655 | 112,509 | 39,130,036 | 348 | 99.03 | 96.82 | 52.36 | 93.11 |
|  | KGR2 | 130,320 | 116,990 | 113,536 | 107,840 | 37,565,673 | 348 | 99.02 | 96.6 | 57.09 | 82.75 |
|  | KL1 | 132,452 | 111,441 | 103,661 | 96,999 | 31,595,967 | 326 | 99.08 | 96.94 | 55.67 | 73.23 |
| H1 | MKCW | 136,415 | 99,343 | 86,734 | 79,534 | 25,824,787 | 325 | 99.16 | 97.18 | 55.06 | 58.3 |
|  | MKE | 128,632 | 124,807 | 123,596 | 123,251 | 42,864,223 | 348 | 99.08 | 96.9 | 52.36 | 95.82 |
|  | MKL2 | 136,136 | 103,732 | 92,573 | 91,490 | 31,754,540 | 347 | 99.08 | 96.95 | 52.32 | 67.2 |
|  | MKLI | 136,865 | 119,657 | 114,499 | 94,078 | 29,570,609 | 314 | 99.05 | 97.07 | 53.37 | 68.74 |
|  | MKPC | 122,537 | 108,018 | 104,027 | 103,645 | 36,044,445 | 348 | 99.09 | 96.96 | 52.39 | 84.58 |
|  | MKP | 106,404 | 93,737 | 89,647 | 88,706 | 30,903,189 | 348 | 99.08 | 96.94 | 52.92 | 83.37 |
| H2 | SJCO | 113,274 | 95,828 | 80,160 | 59,741 | 20,672,369 | 346 | 98.97 | 96.56 | 55.52 | 52.74 |
|  | SJCW | 139,517 | 129,748 | 126,345 | 126,288 | 43,954,254 | 348 | 99.08 | 96.92 | 52.34 | 90.52 |
|  | SJL1 | 133,449 | 106,940 | 97,108 | 96,267 | 33,496,480 | 348 | 99.08 | 96.93 | 52.39 | 72.14 |
|  | SJL2 | 130,398 | 126,930 | 126,020 | 125,605 | 43,801,273 | 349 | 99.08 | 96.89 | 52.4 | 96.32 |

**Data Rarefaction**

Rarefaction curves were used for indicating the biodiversity of the samples in the present study [Lundberg et al., 2013]. These curves were created by randomly selecting certain amount of sequencing data from the samples, and counting the number of the species they represented (i.e., the number of OTUs). The Rarefaction curves directly reflected the rationality of the sequencing data volume and the richness of microbial community in the samples. As the curves became flatter, this assured us that a credible number of samples have been taken. The fig S1 and S2 represent the rarefaction curves for bacterial and fungal sequences respectively. The Goods coverage for the dataset was >99%


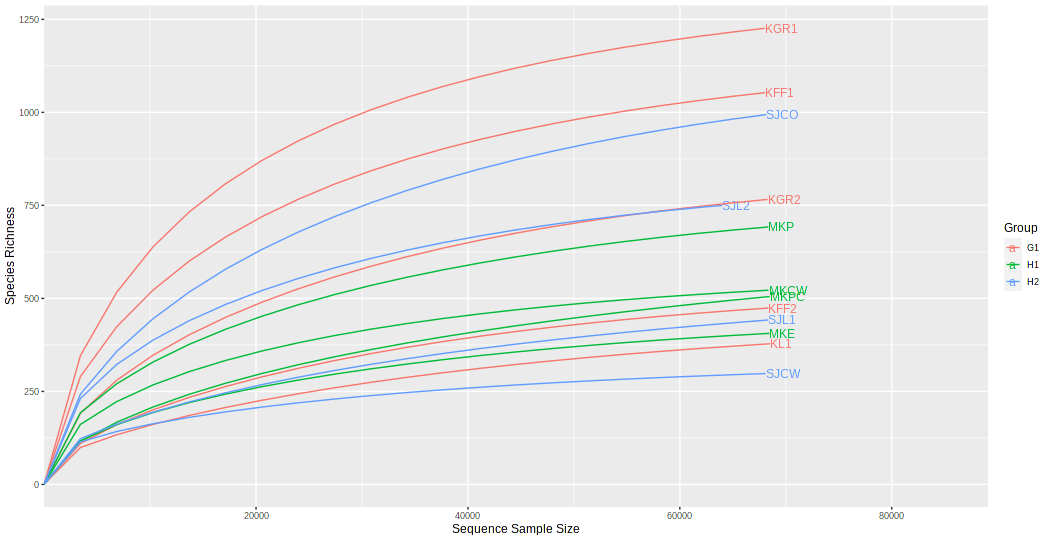

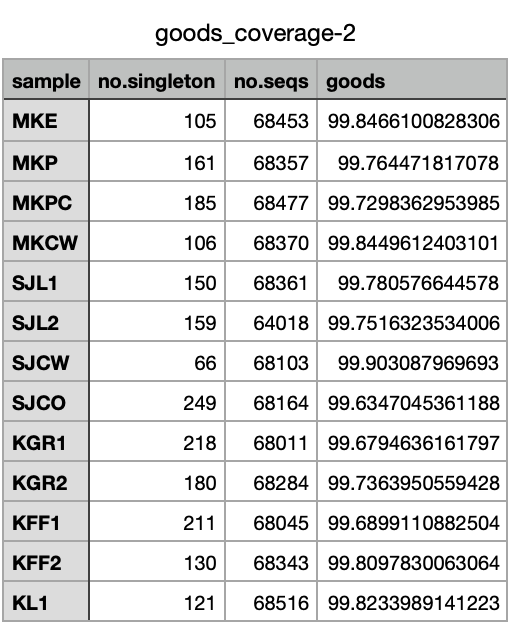


**Fig S1: Data rarefaction Curves for Bacterial libraries**


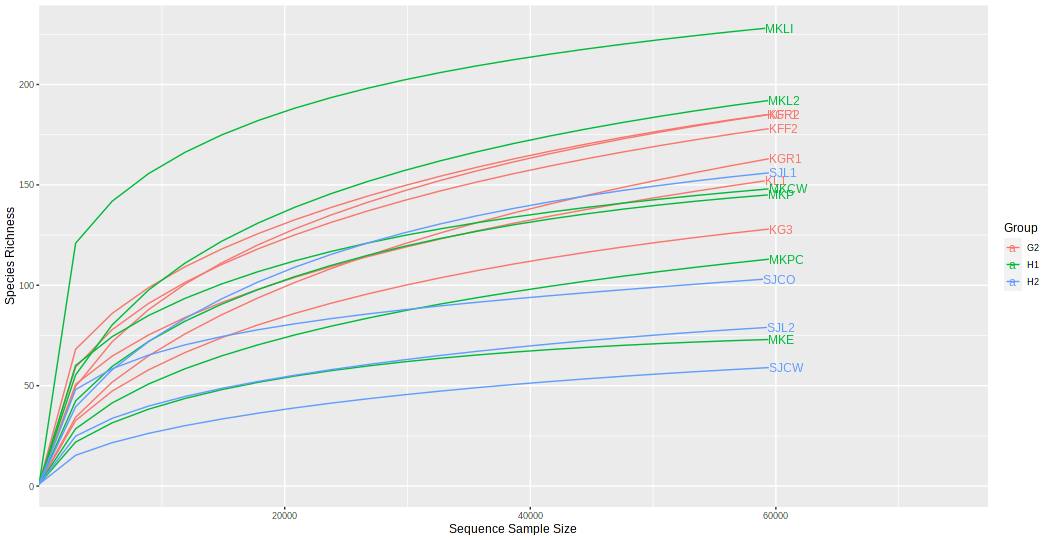

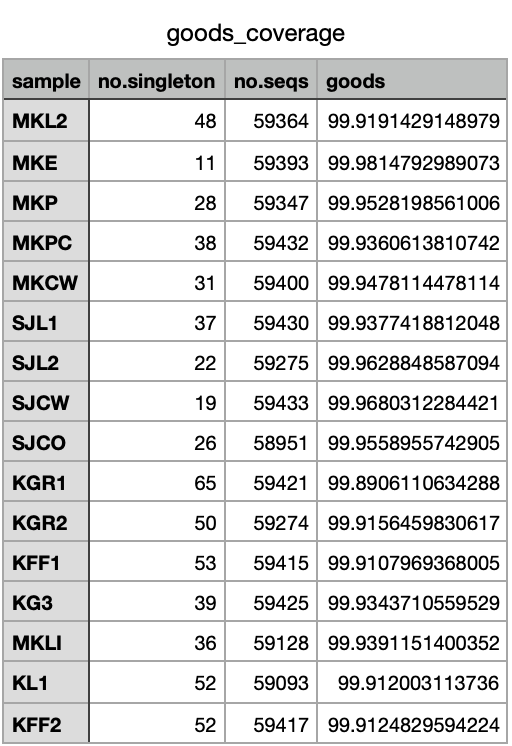


**Fig S2: Data Rarefaction curves for Fungal libraries**

**OTU Analysis**

In order to study the microbial community composition in each sample, Operational Taxonomic Units (OTUs) were obtained by clustering with 97% identity on the Effective Tags of all samples, and then identified by alignment in the SILVA database [Caporaso et al 2010]. In the process of constructing OTUs, basic information of different samples was collected, such as Effective Tags data, low-frequency Tags data and Tags annotation data. The summarization for bacterial and fungal OTUs are shown in figs S3 and S4.

**Fig S3: Bacterial OTU identification**

**Fig S4: Fungal OTU identification**

The Y1-axis titled "Tags Number" means the number of tags;

Total tags"(Red bars) means the number of effective tags;

Taxon Tags" (Blue bars) means the number of annotated tags;

Unclassified Tags" (Green bars) means the number of unannotated tags;

Unique Tags" (Orange bars) means the number of tags with a frequency of 1 and only occurs in one sample.

The Y2-axis titled "OTUs Numbers" means the number of OTUs which displayed as "OTUs" (Purple bars) in the above picture to identify the numbers of OTUs in different samples.

**Taxonomic Profiling**

The bacterial and fungal OTU files can be accessed through the following links:

Bacterial taxonomies

**Table S3: Bacterial OTUs**

<https://docs.google.com/spreadsheets/d/1H7IkNJsmjqQPWyd9gbAty0Ky56MXHxxI/edit?usp=sharing&ouid=115538042225509769286&rtpof=true&sd=true>

Fungal taxonomies

**Table S4: Fungal OTUs**

<https://docs.google.com/spreadsheets/d/1gDC_uAvuh5uMnQSGNm3llJSWeoi9uFrB/edit?usp=sharing&ouid=115538042225509769286&rtpof=true&sd=true>

**Intra-Site variability**

**Table S5: RA of top ten bacterial genera within the sublocations of H1, H2 and G2**

|  | **MKE** | **MKP** | **MKPC** | **MKCW** |  | **SJL1** | **SJL2** | **SJCW** | **SJCO** |  | **KGR1** | **KGR2** | **KFF1** | **KFF2** | **KL1** |
| --- | --- | --- | --- | --- | --- | --- | --- | --- | --- | --- | --- | --- | --- | --- | --- |
| **Taonella** | 0.322 | 0.302 | 0.006 | 0.013 |  | 0.001 | 0.000 | 0.009 | 0.009 |  | 0.032 | 0.015 | 0.001 | 0.007 | 0.014 |
| **Sphingobium** | 0.137 | 0.150 | 0.046 | 0.025 |  | 0.004 | 0.001 | 0.047 | 0.013 |  | 0.041 | 0.040 | 0.002 | 0.021 | 0.040 |
| **Sphingopyxis** | 0.070 | 0.053 | 0.045 | 0.019 |  | 0.011 | 0.001 | 0.028 | 0.018 |  | 0.032 | 0.015 | 0.001 | 0.008 | 0.016 |
| **Phenylobacterium** | 0.066 | 0.081 | 0.007 | 0.009 |  | 0.001 | 0.001 | 0.017 | 0.011 |  | 0.012 | 0.024 | 0.001 | 0.001 | 0.000 |
| **Variovorax** | 0.042 | 0.038 | 0.098 | 0.068 |  | 0.022 | 0.005 | 0.049 | 0.061 |  | 0.276 | 0.230 | 0.018 | 0.219 | 0.074 |
| **Bradyrhizobium** | 0.013 | 0.016 | 0.019 | 0.018 |  | 0.003 | 0.002 | 0.014 | 0.034 |  | 0.021 | 0.049 | 0.004 | 0.006 | 0.009 |
| **Actinobacillus** | 0.011 | 0.003 | 0.000 | 0.008 |  | 0.000 | 0.005 | 0.021 | 0.017 |  | 0.028 | 0.017 | 0.023 | 0.007 | 0.000 |
| **Sphingomonas** | 0.010 | 0.011 | 0.024 | 0.006 |  | 0.004 | 0.001 | 0.020 | 0.006 |  | 0.030 | 0.009 | 0.008 | 0.002 | 0.010 |
| **Methylibium** | 0.009 | 0.012 | 0.018 | 0.010 |  | 0.007 | 0.003 | 0.039 | 0.046 |  | 0.154 | 0.073 | 0.002 | 0.011 | 0.017 |
| **Parvibaculum** | 0.008 | 0.003 | 0.229 | 0.154 |  | 0.043 | 0.010 | 0.183 | 0.197 |  | 0.052 | 0.098 | 0.004 | 0.022 | 0.043 |

**Table S6: RA of top ten fungal species within the sublocations of H1, H2 and G2**

|  | **MKL2** | **MKE** | **MKP** | **MKPC** | **MKCW** | **MKLI** |  | **SJL1** | **SJL2** | **SJCW** | **SJCO** |  | **KGR1** | **KGR2** | **KFF1** | **KG3** | **KL1** | **KFF2** |
| --- | --- | --- | --- | --- | --- | --- | --- | --- | --- | --- | --- | --- | --- | --- | --- | --- | --- | --- |
| ***Wilcoxina rehmii*** | 0.972 | 0.992 | 0.826 | 0.985 | 0.077 | 0.055 |  | 0.976 | 0.979 | 0.996 | 0.325 |  | 0.988 | 0.029 | 0.862 | 0.976 | 0.003 | 0.013 |
| ***Penicillium desertorum*** | 0.004 | 0.001 | 0.001 | 0.002 | 0.530 | 0.008 |  | 0.001 | 0.001 | 0.000 | 0.002 |  | 0.000 | 0.076 | 0.004 | 0.000 | 0.006 | 0.000 |
| ***Saccharomyces cerevisiae*** | 0.004 | 0.000 | 0.006 | 0.000 | 0.001 | 0.019 |  | 0.001 | 0.000 | 0.000 | 0.001 |  | 0.001 | 0.000 | 0.000 | 0.000 | 0.007 | 0.000 |
| ***Aspergillus ruber*** | 0.002 | 0.000 | 0.001 | 0.004 | 0.076 | 0.036 |  | 0.001 | 0.007 | 0.000 | 0.270 |  | 0.001 | 0.367 | 0.003 | 0.011 | 0.741 | 0.003 |
| ***Humicola grisea*** | 0.002 | 0.001 | 0.001 | 0.001 | 0.007 | 0.535 |  | 0.002 | 0.001 | 0.000 | 0.000 |  | 0.000 | 0.001 | 0.000 | 0.000 | 0.000 | 0.001 |
| ***Heterophoma sylvatica*** | 0.001 | 0.000 | 0.000 | 0.000 | 0.000 | 0.088 |  | 0.001 | 0.000 | 0.000 | 0.000 |  | 0.000 | 0.000 | 0.000 | 0.000 | 0.000 | 0.000 |
| ***Malassezia restricta*** | 0.000 | 0.000 | 0.000 | 0.000 | 0.028 | 0.002 |  | 0.000 | 0.000 | 0.000 | 0.006 |  | 0.000 | 0.017 | 0.001 | 0.000 | 0.070 | 0.000 |
| ***Colletotrichum gloeosporioides*** | 0.000 | 0.000 | 0.000 | 0.000 | 0.000 | 0.013 |  | 0.000 | 0.000 | 0.000 | 0.000 |  | 0.000 | 0.000 | 0.000 | 0.000 | 0.000 | 0.001 |
| ***Fusarium proliferatum*** | 0.000 | 0.000 | 0.000 | 0.000 | 0.000 | 0.052 |  | 0.001 | 0.000 | 0.000 | 0.013 |  | 0.002 | 0.003 | 0.003 | 0.002 | 0.002 | 0.003 |
| ***Leptobacillium leptobactrum*** | 0.000 | 0.000 | 0.000 | 0.000 | 0.000 | 0.000 |  | 0.005 | 0.000 | 0.000 | 0.000 |  | 0.001 | 0.045 | 0.002 | 0.001 | 0.049 | 0.418 |

**Alpha Diversity Analysis**

Alpha diversity was applied in analyzing complexity of biodiversity for a sample through 6 indices, including Observed-species, Chao1, Shannons, Simpsons, ACE, Good-coverage. All these indices in our samples were calculated with QIIME (Version 1.7.0) and displayed with R software (Version 2.15.3).

**Table S7: Alpha Diversity Indices of Bacterial Species**

|  | **Hospital** | **Group** | **samples** | **Observed** | **Chao1** | **ACE** | **Shannon** | **Simpson** | **Fisher** | **Goods Coverage** | **PD whole tree** |
| --- | --- | --- | --- | --- | --- | --- | --- | --- | --- | --- | --- |
| **1** | MKH | H1 | MKE | 142 | 145 | 146 | 3 | 0.854 | 17 | 0.998 | 46.210 |
| **2** | MKH | H1 | MKP | 184 | 185 | 187 | 3 | 0.861 | 23 | 0.997 | 63.295 |
| **3** | MKH | H1 | MKPC | 159 | 173 | 171 | 3 | 0.890 | 20 | 0.997 | 50.667 |
| **4** | MKH | H1 | MKCW | 173 | 178 | 178 | 3 | 0.921 | 22 | 0.998 | 71.440 |
| **5** | SJH | H2 | SJL1 | 135 | 147 | 146 | 3 | 0.906 | 16 | 0.998 | 46.420 |
| **6** | SJH | H2 | SJL2 | 194 | 200 | 199 | 1 | 0.440 | 25 | 0.997 | 197.537 |
| **7** | SJH | H2 | SJCW | 109 | 118 | 116 | 3 | 0.940 | 13 | 0.999 | 44.305 |
| **8** | SJH | H2 | SJCO | 218 | 225 | 222 | 3 | 0.917 | 28 | 0.996 | 111.310 |
| **9** | KISR | G1 | KGR1 | 215 | 216 | 216 | 3 | 0.887 | 28 | 0.998 | 102.862 |
| **10** | KISR | G1 | KGR2 | 205 | 207 | 209 | 3 | 0.911 | 26 | 0.998 | 90.629 |
| **11** | KISR | G1 | KFF1 | 199 | 202 | 201 | 3 | 0.900 | 26 | 0.997 | 105.777 |
| **12** | KISR | G1 | KFF2 | 142 | 145 | 147 | 2 | 0.661 | 17 | 0.997 | 54.259 |
| **13** | KISR | G1 | KL1 | 140 | 143 | 145 | 2 | 0.821 | 17 | 0.997 | 43.532 |

**Table S8: Alpha Diversity Indices of Fungal species**

|  | **Hospital** | **Group** | **samples** | **Observed** | **CHao 1** | **ACE** | **Shannon’s** | **Simpson** | **Fisher** | **Goods Coverage** | **PD whole tree** |
| --- | --- | --- | --- | --- | --- | --- | --- | --- | --- | --- | --- |
| **1** | MKH | H1 | MKL2 | 29 | 29 | 29 | 0.196 | 0.054 | 3 | 0.999 | 79.601 |
| **2** | MKH | H1 | MKE | 24 | 25 | 25 | 0.061 | 0.015 | 2 | 0.999 | 69.185 |
| **3** | MKH | H1 | MKP | 30 | 31 | 32 | 0.565 | 0.294 | 3 | 1 | 32.091 |
| **4** | MKH | H1 | MKPC | 27 | 28 | 29 | 0.109 | 0.029 | 3 | 0.999 | 58.066 |
| **5** | MKH | H1 | MKCW | 30 | 32 | 34 | 1.375 | 0.635 | 3 | 0.999 | 37.954 |
| **6** | SJH | H2 | SJL1 | 33 | 34 | 35 | 0.169 | 0.047 | 3 | 0.999 | 57.089 |
| **7** | SJH | H2 | SJL2 | 23 | 24 | 25 | 0.135 | 0.042 | 2 | 0.999 | 48.762 |
| **8** | SJH | H2 | SJCW | 19 | 19 | 21 | 0.032 | 0.008 | 2 | 1 | 29.87 |
| **9** | SJH | H2 | SJCO | 21 | 22 | 22 | 1.602 | 0.760 | 2 | 1 | 29.389 |
| **10** | KISR | G2 | KGR1 | 35 | 42 | 41 | 0.093 | 0.024 | 4 | 1 | 32.623 |
| **11** | KISR | G2 | KGR2 | 30 | 31 | 31 | 1.595 | 0.715 | 3 | 0.999 | 46.432 |
| **12** | KISR | G2 | KFF1 | 35 | 38 | 37 | 0.512 | 0.243 | 4 | 0.999 | 51.837 |
| **13** | KISR | G2 | KG3 | 27 | 29 | 29 | 0.153 | 0.048 | 3 | 0.999 | 55.097 |
| **14** | MKH | H1 | MKLI | 32 | 33 | 34 | 1.855 | 0.691 | 3 | 0.999 | 79.601 |
| **15** | KISR | G2 | KL1 | 31 | 34 | 33 | 0.743 | 0.308 | 3 | 0.999 | 50.073 |
| **16** | KISR | G2 | KFF2 | 30 | 32 | 31 | 1.212 | 0.659 | 3 | 0.999 | 38.952 |

**Beta Diversity Analysis**

Beta diversity analysis was used to evaluate differences of samples in species complexity, Beta diversity on both weighted and unweighted unifrac were calculated by QIIME software (Version 1.7.0). Cluster analysis was preceded by principal component analysis (PCA), which was applied to reduce the dimension of the original variables using the FactoMineR package and ggplot2 package in R software (Version 2.15.3). Principal Coordinate Analysis (PCoA) was performed to get principal coordinates and visualize from complex, multidimensional data. A distance matrix of weighted or unweighted unifrac among samples obtained before was transformed to a new set of orthogonal axes, by which the maximum variation factor is demonstrated by first principal coordinate, and the second maximum one by the second principal coordinate, and so on. PCoA analysis was displayed by WGCNA package, stat packages and ggplot2 package in R software (Version 2.15.3). Unweighted Pair-group Method with Arithmetic Means (UPGMA) Clustering was performed as a type of hierarchical clustering method to interpret the distance matrix using average linkage and was conducted by QIIME software (Version 1.7.0) [Lozupone et al., 2007].


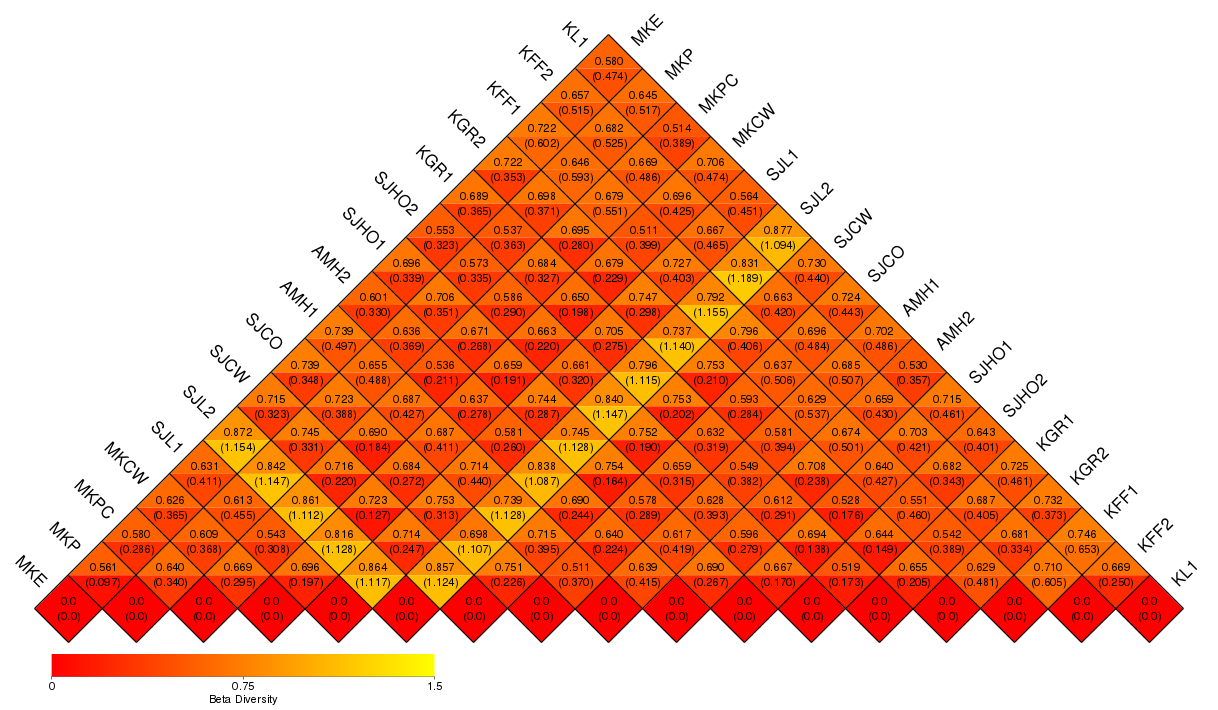


**Fig S5: Beta diversity analysis on Unweighted UniFrac distances of bacterial phyla**


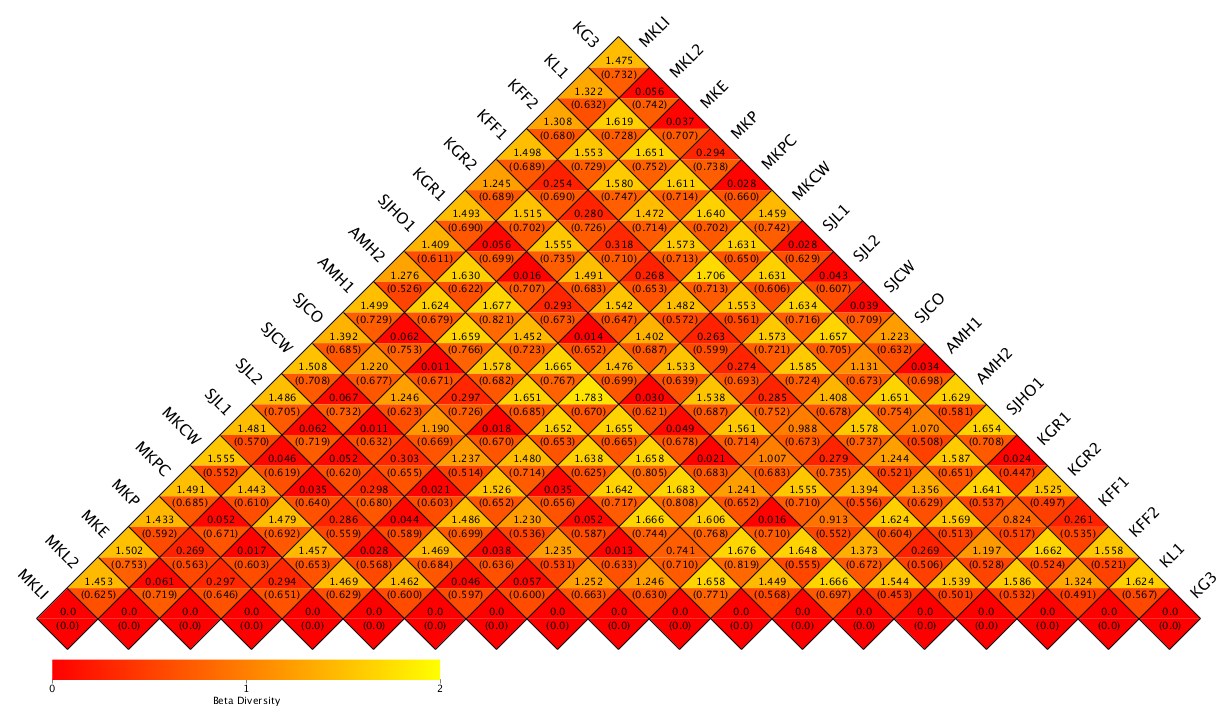


**Fig S6: Beta diversity analysis on Unweighted UniFrac distances of fungal phyla**

**Functional Prediction**

**Table S9 KEGG Ontologies associated with Bacterial genera**

|  | **H1** | **G2** | **H2** |
| --- | --- | --- | --- |
| **Amino acid metabolism** | 1241682 | 259358 | 596820 |
| **Biosynthesis of other secondary metabolites** | 104454 | 21157 | 51772 |
| **Carbohydrate metabolism** | 1182550 | 236549 | 599558 |
| **Energy metabolism** | 821294 | 170773 | 391455 |
| **Glycan biosynthesis and metabolism** | 191446 | 36524 | 101093 |
| **Lipid metabolism** | 380016 | 78790 | 185686 |
| **Metabolism of cofactors and vitamins** | 698588 | 145830 | 335793 |
| **Metabolism of other amino acids** | 377595 | 78679 | 182341 |
| **Metabolism of terpenoids and polyketides** | 207957 | 42707 | 102279 |
| **Nucleotide metabolism** | 540267 | 109193 | 277530 |
| **Xenobiotics biodegradation and metabolism** | 285562 | 61611 | 132016 |

**References**

Caporaso, J. Gregory, et al. Global patterns of 16S rRNA diversity at a depth of millions of sequences per sample. Proceedings of the National Academy of Sciences 108.Supplement 1 (2011): 4516-4522.

Ondov, Brian D., Nicholas H. Bergman, and Adam M. Phillippy. Interactive metagenomic visualization in a Web browser. BMC bioinformatics 12.1 (2011): 385.

Caporaso, J. Gregory, et al. QIIME allows analysis of high-throughput community sequencing data. Nature methods 7.5 (2010): 335-336.

Lundberg, Derek S., et al. Practical innovations for high-throughput amplicon sequencing.Nature methods 10.10 (2013): 999-1002.

Lozupone, Catherine A., et al. Quantitative and qualitative β diversity measures lead to different insights into factors that structure microbial communities. Applied and environmental microbiology 73.5 (2007): 1576-1585.
